# ChanFAD: A Functional Annotation Database for Ion Channels

**DOI:** 10.3389/fbinf.2022.835805

**Published:** 2022-07-25

**Authors:** Elizabeth V. Castro, John W. Shepherd, Ryan S. Guggenheim, Manimone Sengvoravong, Bailey C. Hall, McKenzie K. Chappell, Jessica A. Hearn, Olivia N. Caraccio, Cora Bissman, Sydney Lantow, Damian Buehner, Harry R. Costlow, David M. Prather, Abigail M. Zonza, Mallory Witt, Jeffrey A. Zahratka

**Affiliations:** ^1^ Department of Neuroscience, Baldwin Wallace University, Berea, OH, United States; ^2^ Department of Psychology, Baldwin Wallace University, Berea, OH, United States; ^3^ Department of Biology and Geology, Baldwin Wallace University, Berea, OH, United States; ^4^ Department of Chemistry, Baldwin Wallace University, Berea, OH, United States

**Keywords:** ion channels, functional annotation, protein structure, database, proteomics

## Abstract

Ion channels are integral membrane protein complexes critical for regulation of membrane potential, cell volume, and other signaling events. As complex molecular assemblies with many interacting partners, ion channels have multiple structural and functional domains. While channel sequence and functional data are readily available across multiple online resources, there is an unmet need for functional annotation directly relating primary sequence information, 2D interactions, and three-dimensional protein structure. To this end, we present ChanFAD (Channel Functional Annotation Database), to provide the research community with a centralized resource for ion channel structure and functional data. ChanFAD provides functional annotation of PDB structures built on the National Center for Biotechnology Information’s iCn3D structure viewing tool while providing additional information such as primary sequence, organism, and relevant links to other databases. Here we provide a brief tour of ChanFAD functionality while showing example use cases involving drug-channel interactions and structural changes based on mutation. ChanFAD is freely available and can be accessed at https://www.chanfad.org/.

## Introduction

Ion channels are a broad family of integral membrane proteins characterized by the ability to transport ions and other small molecules across the phospholipid bilayer. Ubiquitously expressed and evolutionarily ancient, ion channels are critical regulators of cell volume, membrane potential, and gene expression (Berridge, 2014). Structurally, ion channels are made from multiple protein subunits that integrate into complex assemblies of scaffold proteins, enzymes, and other regulators to mediate localized intracellular signaling (Catterall, 2010; Dai et al., 2009). As multi-subunit protein complexes, ion channels have multiple structural and functional domains necessary for transmembrane localization, post-translational modification, and protein-protein interactions giving rise to channel function. Studying ion channel structure, function, and regulation has been a rewarding endeavor in diverse biological disciplines, but there is a notable lack of detailed annotation data mapping functional domains and post-translational modification sites to three-dimensional protein structure. To address this need, we introduce the Channel Functional Annotation Database (ChanFAD), a centralized community resource for relating genomic, biophysical, and bioinformatic information to map ion channel function to three-dimensional protein structure. ChanFAD entries are manually curated using a combination of primary literature, previously validated annotations, and available PDB structures in the National Center for Biotechnology Information’s iCn3D model structure viewer (Wang et al., 2020). Importantly, ChanFAD aims to build upon known annotation information found in general resources such as Pfam and Gene Ontology by focusing on functional domains specific to each ion channel complex in more detail. Each ChanFAD page contains the annotated PDB structure displayed in iCn3D accompanied by a downloadable selection file for external use, a link to the primary sequence in FASTA format, a “two-dimensional” representation of functional domains using the Swiss Institute of Bioinformatics neXtProt feature viewer, and links to relevant databases related to ion channel structure, function, or both. Specifically, ChanFAD provides links to the structural databases of RCSB PDB, UniProt, and predicted structures from AlphaFold; functional data from AmiGO. InterPro, and KEGG; and ion channel-specific databases of Channelpedia and IUPHAR entries for a given protein (Mao et al., 2005; Carbon et al., 2009, 2021; Blum et al., 2021; Jumper et al., 2021; Harding et al., 2022; Varadi et al., 2022). Additionally, the ChanFAD website provides links to other relevant resources to the ion channel community not specific to individual channel complexes, such as computational models provided by ModelDB and Ion Channel Genealogy, HOLE and ChannelsDB for exploring channel pore structure, and more generalized databases such as Pfam and the Neuroscience Information Framework (Smart et al., 1993; Gardner et al., 2008; McDougal et al., 2017; Podlaski et al., 2017; Pravda et al., 2018; Mistry et al., 2021). By providing functional annotation information for ion channels and grouping relevant links together, we envision multidisciplinary teams, including geneticists, structural biologists, and electrophysiologists, collaboratively working together to decipher ion channel structure-function relationships. Our aim is to provide an intuitive interface for bridging the gap between the 3D structural properties often studied by biophysicists and electrophysiologists with the functional domains probed by geneticists and molecular biologists. Here, we briefly detail the annotation process and demonstrate three examples of ChanFAD functionality highlighting the importance of structure-function relationships in ion channel activity and disease.

## Description

ChanFAD aims to be a comprehensive resource for ion channel functional annotation data. The defining feature of the database is its annotations, which are manually curated from a combination of previously annotated functional domains found on Pfam and NCBI’s Protein database, existing iCn3D data, and primary literature. ChanFAD annotations are represented in iCn3D selection sets, which allow for convenient 3D representation of a given domain. Annotations are created by finding an ion channel with a PDB structure on the RCSB PDB database (Berman, 2000). Next, the curator performs a thorough literature search, looking for descriptions of other domains not previously represented. Each domain is then organized and mapped to a PDB file using iCn3D’s Defined Sets feature, which is then freely available as part of an embedded iCn3D instance for the public on ChanFAD. The annotation process is then replicated for each unique subunit found in the PDB file, when applicable. One potential limitation of long annotations is the iCn3D shared link limit of 4,000 characters. As large protein complexes can easily result in detailed annotations that generate long links, we made the decision to annotate only the largest subunits of the PDB file when our annotations reach this limit, and only label each unique protein once, even if multiple identical chains are present. However, extremely large channels such as ryanodine receptors still exceed this limit, and possibilities for full annotation are currently being implemented for visualization.

The initial release of ChanFAD is built on the web using the Django framework (v2.1.2) in Python (v3.9.9) and can be accessed at https://www.chanfad.org/.

## Example Annotations

**FIGURE 1:**
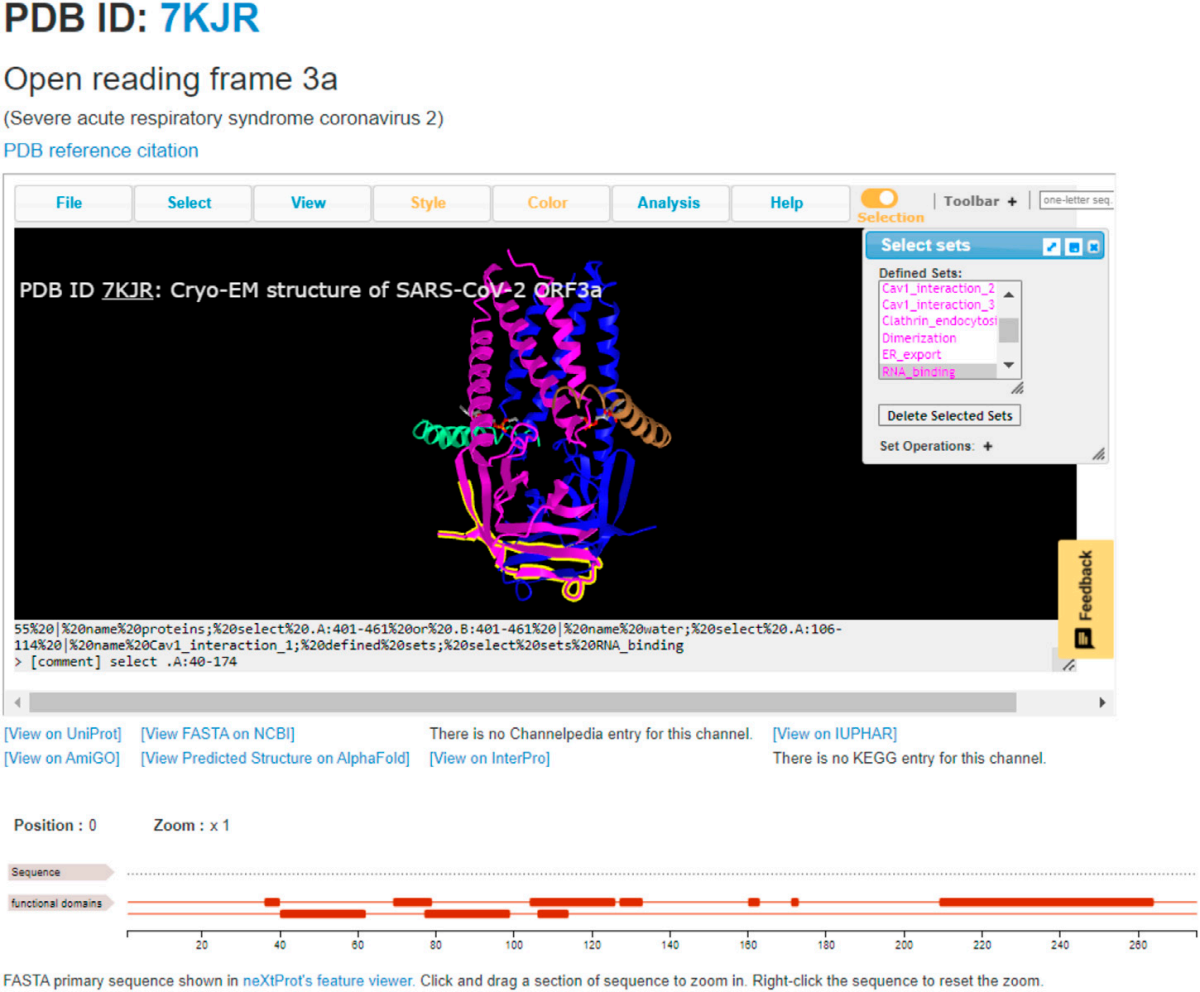
The annotated SARS-CoV-2 open reading frame 3a ion channel (PDB ID: 7KJR) with RNA-binding domain highlighted. This domain is the cytoplasmic side of the cell membrane, where it may be involved in binding viral RNA and apoptosis.

The most straightforward usage of ChanFAD is simple viewing of annotated domains, which can be especially useful for studying emerging topics or mapping genetic regions of interest onto a 3D protein structure. The example we will cover in this manuscript is the open reading frame 3a viral ion channel from SARS-CoV-2, the causative agent of COVID-19 (PDB ID: 7KJR). While ORF3a function is still being characterized at the time of writing, one of the channel’s possible quaternary structures has been solved using cryo-electron microscopy in lipid nanodiscs to reveal a peculiar homodimer configuration (Kern et al., 2020). While the initial RCSB entry shows a SARS-CoV-like ORF3a-like domain covering the entire length of each protein subunit, further exploration of the literature demonstrates multiple functional domains, including those for caveolin-1 interaction, clathrin-mediated endocytosis, RNA-binding, and TRAF3-binding ultimately resulting in recruitment of the NLRP3 inflammasome potentially resulting in apoptosis (Hassan et al., 2020; Issa et al., 2020; Ren et al., 2020). Each of these domains are represented and available to view in ChanFAD, as in the example screenshot shown in Figure 1. Also notable in the screenshot are direct links to the RCSB PDB database (by clicking on the PDB ID), reference to the publication characterizing the structure, UniProt entry, primary sequence in FASTA format, structure prediction using AlphaFold, and entries on AmiGO and InterPro. Notably, links for Channelpedia, KEGG, and IUPHAR are not available as information on ORF3a structure and function is still emerging.

**FIGURE 2:**
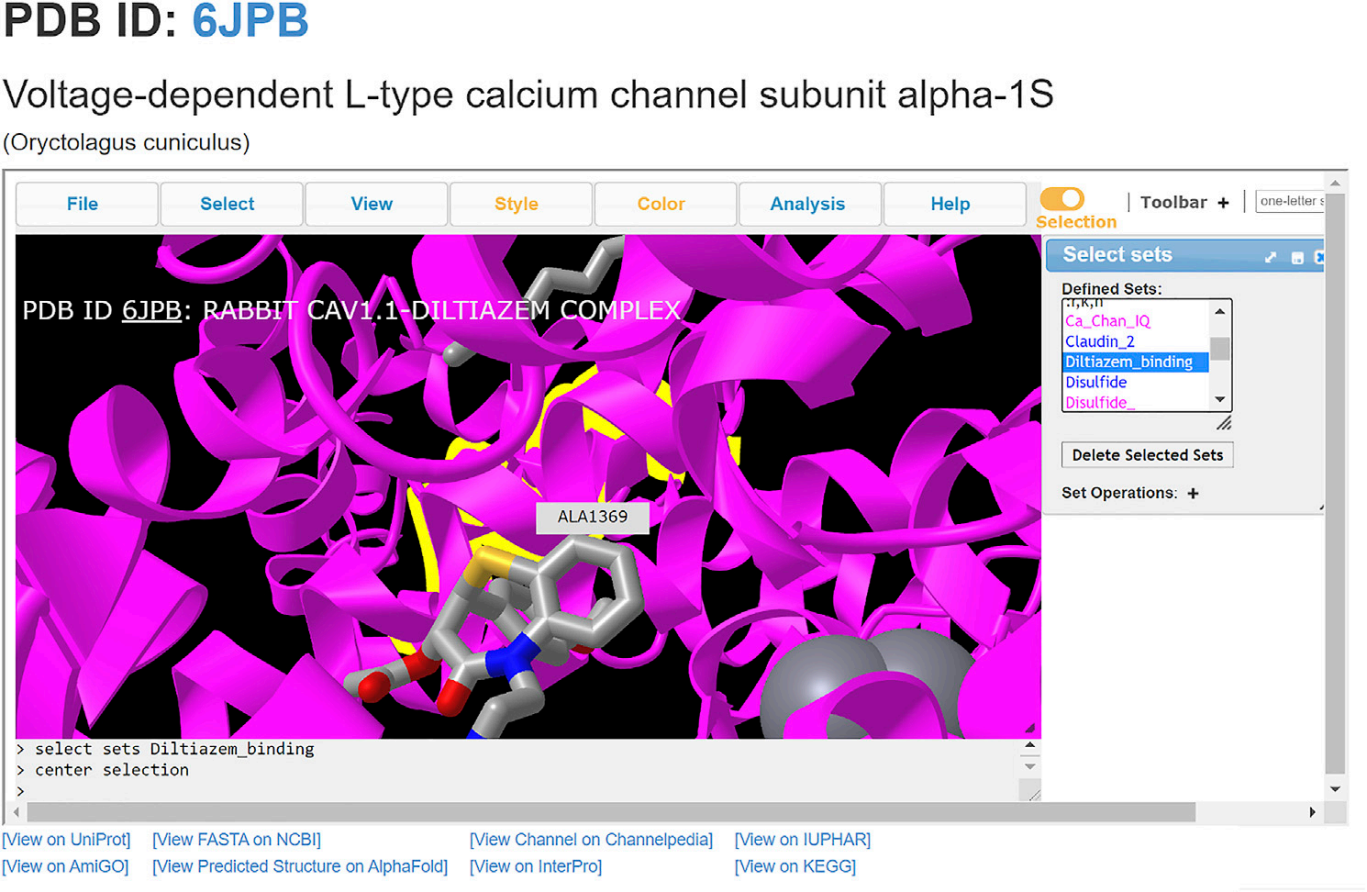
Visualization of diltiazem blocking the Ca_V_1.1 channel pore (PDB: 6JPB). Diltiazem interacts with three conserved residues (Y1365, A1369, and I1372) on the intracellular side of the channel to block calcium conductance. Ca^2+^ ions shown as gray spheres. The alanine residue (A1369) is centered, with the diltiazem molecule visibly blocking the channel pore.

ChanFAD can also leverage the strength of iCn3D’s viewing capabilities for easy visualization of ligand-channel interactions. In this example, we will examine the binding of the rabbit (*Oryctolagus cuniculus*) voltage-gated calcium channel alpha-1 subunit (Ca_V_1.1) with the benzothiazepine pore blocker diltiazem (PDB ID: 6JPB) (Zhao et al., 2019). Diltiazem has a straightforward mechanism of action: it physically blocks the channel pore on the intracellular side of the selectivity filter, disrupting ion flow (Tang et al., 2019). Structurally, the selectivity filter is a set of amino acids in the pore-forming domains of voltage-gated ion channels which confer ion specificity to the channels (Jiang et al., 2002). Annotation of the critical residues for diltiazem binding (Y1365, A1369, and I1372) are present in ChanFAD, and can be visualized in the iCn3D panel for view along with the previously mentioned RCSB PDB and Channelpedia database links (Figure 2) (Zhao et al., 2019). In contrast to the ORF3a entry discussed in Figure 1, the 6JPB entry shown in Figure 2 does have available links for the Channelpedia, KEGG, and IUPHAR databases. We foresee having this information in a single entry will facilitate studies of mapping genetic mutations to channel function both in research and classroom settings.

**FIGURE 3:**
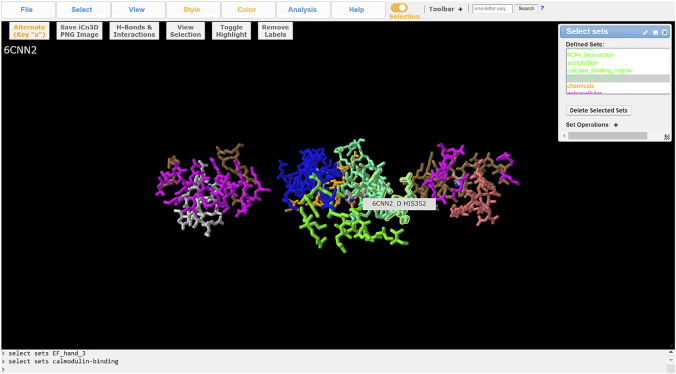
Visualization of the SK4 calcium-activated potassium channel (PDB ID: 6CNN) in iCn3D with ChanFAD annotation and the R352H mutation introduced using scap. R352H is in the calmodulin interaction domain, which is responsible for calcium binding and channel opening. The figure only shows the four calmodulin binding domains (one for each protein subunit) for readability.

ChanFAD annotations can also be leveraged with iCn3D’s full capabilities for visualizing mutations. One powerful capability of iCn3D is the ability to make point mutations to PDB structures and render them in the viewer using scap (Jacobson et al., 2002; Xiang & Honig, 2001). Although ChanFAD automatically loads annotation files directly into iCn3D when the database is accessed, the annotation files are available for public download for further downstream analysis. For example, we will look at mutation of a human calcium-gated potassium channel subunit (SK4, PDB ID: 6CNN) where specific point mutations can result in dehydrated hereditary stomatocytosis type 2 (Lee & MacKinnon, 2018). Affected patients have a heterozygous substitution in a conserved calmodulin interaction domain (R352H), resulting in increased sensitivity to calcium ions and frequent channel opening resulting in altered red blood cell shape, cellular fragility, and anemia (Rapetti-Mauss et al., 2015). First, we obtain the mutated PDB by using iCn3D’s built-in mutation function (Analysis → Mutation) and specifying the R352H mutation (e.g., 6CNN_A_352_H) and opening a new iCn3D browser instance. Loading the ChanFAD annotation into the mutated PDB, we can highlight the calmodulin binding domain and see the change in the protein side chain, permitting visualization of structural changes for downstream analysis (Figure 3). In this way, we envision ChanFAD annotations can be used in tandem with shareable iCn3D links and PDB files for reproducible structural studies of ion channels.

## Future Directions

As a database of manual annotations, the primary future direction of ChanFAD is the addition of new entries available for view. However, there are a few other features our team would like to add in the future. First, providing a table of annotated domains with associated references would be a valuable reference for structural and genetic studies, even if the full three-dimensional representation found in iCn3D is not needed. Second, we wish to add more robust literature curation to each database entry to help researchers and integrate peer-reviewed experiments with structural and functional domains, which synergizes well with the tabular representation of annotated domains mentioned previously. Third, we are actively working to implement iCn3D functionality for large annotations resulting in shared links of greater than 4,000 characters. One potential solution is the use of iCn3D PNG files or state files, which should be integrated into ChanFAD at a later date. We are also exploring the possibility of using natural language processing methods, such as BioBERT, to perform text mining on published literature to speed up the annotation process (Lee et al., 2020). Finally, as a community resource, we are working to implement both a community submission process for new annotation data through our Django-based web framework which would then be reviewed for accuracy and made available, and a convenient and secure way to directly load the annotation files from ChanFAD directly into other applications. Our ultimate goal is to easily facilitate collaborative research about ion channel structure and function.

## Data Availability

Publicly available datasets were analyzed in this study. This data can be found here: https://github.com/jzahratka/ChanFAD.
